# A Rare Epithelioid Hemangioendothelioma of the Tongue: A Case Report and Review of Published Cases

**DOI:** 10.1155/crid/4933968

**Published:** 2026-05-22

**Authors:** Gioele Gioco, Romeo Patini, Enrico Russo, Guido Rindi, Carlo Lajolo, Cosimo Rupe

**Affiliations:** ^1^ Head and Neck Department, Fondazione Policlinico Universitario A. Gemelli IRCCS Università Cattolica del Sacro Cuore, Rome, 00168, Italy; ^2^ Institute of Pathology, Fondazione Policlinico Universitario “A. Gemelli”, IRCCS, Università Cattolica del Sacro Cuore, Rome, 00168, Italy, unicatt.it

**Keywords:** case report, oral epithelioid hemangioendothelioma, oral medicine, systematic review, vascular neoplasm

## Abstract

**Aim:**

Epithelioid hemangioendothelioma (EHE) is a rare vascular tumor caused by the proliferation of endothelial cells. The purpose of this study was to describe a rare case of EHE of the tongue and to perform a systematic review of the literature reporting oral EHE.

**Methods:**

A 58‐year‐old woman was referred to our Oral Medicine Department for evaluation of a slowly growing ulcerative lesion of the tongue. Her medical history was unremarkable. Intraoral examination revealed a soft ulcerated mass with hypertrophic margins and a yellowish base located on the right lateral border of the tongue, measuring ~2 cm × 3 cm. An incisional biopsy was performed, and the histological exam revealed hyperplastic epithelium, a site of proliferation of epithelioid endothelial elements. Immunohistochemistry showed positivity for ERG, and a final diagnosis of EHE was made. After excisional surgery, no local recurrence was reported at 24 months’ follow‐up. A systematic review was conducted on the PubMed database, following PRISMA guidelines.

**Results:**

Among 35 included studies, 44 cases of EHE were identified, including our case report (21 male and 21 female), with a mean age of 35 years (±19). The most frequently affected site was the gingiva, followed by the tongue (18 and 11). Local recurrence after surgical excision was reported in 18% of cases (8/44). Only one case of metastasis was documented.

**Conclusion:**

Vascular tumors should be considered in the differential diagnosis of soft tissue swellings of the oral cavity. Patients diagnosed with EHE require long‐term follow‐up due to the risk of local recurrence and the tumor’s uncertain malignant potential.

## 1. Introduction

Epithelioid hemangioendothelioma (EHE) is an uncommon, rare vascular neoplasm characterized by proliferation of endothelial cells around the vascular lumen that usually develops into soft tissue and organs (e.g., lung, liver, bone, and musculoskeletal system) with potential for local recurrence and distant metastasis [1, 2].

Weiss and Enzinger [3] first introduced the term EHE to describe a vascular soft tissue neoplasm characterized by the proliferation of endothelial cells with an epithelioid appearance. EHE is an extremely rare vascular tumor with intermediate malignant potential, showing a tendency for local recurrence and, in some cases, distant metastasis [4].

Although its exact etiology remains unclear, epidemiological data estimate a prevalence of ~1 case per 1,000,000 individuals and an annual incidence of about 0.038 per 100,000 population. EHE can occur at any age, including in pediatric patients [5]. However, it is more frequently reported between the second and fifth decades of life, without a clear sex predilection or identifiable predisposing factors [6].

From a clinical perspective, EHE may arise in both superficial and deep soft tissues and has been described in various anatomical locations, including the extremities, lungs, liver, bones, and lymph nodes. In contrast, involvement of the oral cavity is uncommon [7]. Histopathologically, EHE is characterized by nests and cords of epithelioid endothelial cells, often showing cytoplasmic vacuolization within a fibrotic or hyalinized stroma. The lesion may also display proliferation of small capillary‐like vascular channels associated with epithelioid endothelial cells, sometimes intermingled with solid sheets of epithelioid or spindle‐shaped cells [8].

EHE occurring in the oral cavity is rare, and only a limited number of cases have been reported in the literature [9]. Therefore, the aim of the present study was to describe a rare case of EHE arising on the tongue of a 58‐year‐old Italian woman. In addition, a systematic review of the literature was conducted to identify previously reported cases of oral EHE.

## 2. Methods

### 2.1. Case Report

The present case report was prepared in accordance with the CARE Guidelines (File S1).

**Figure 1:**
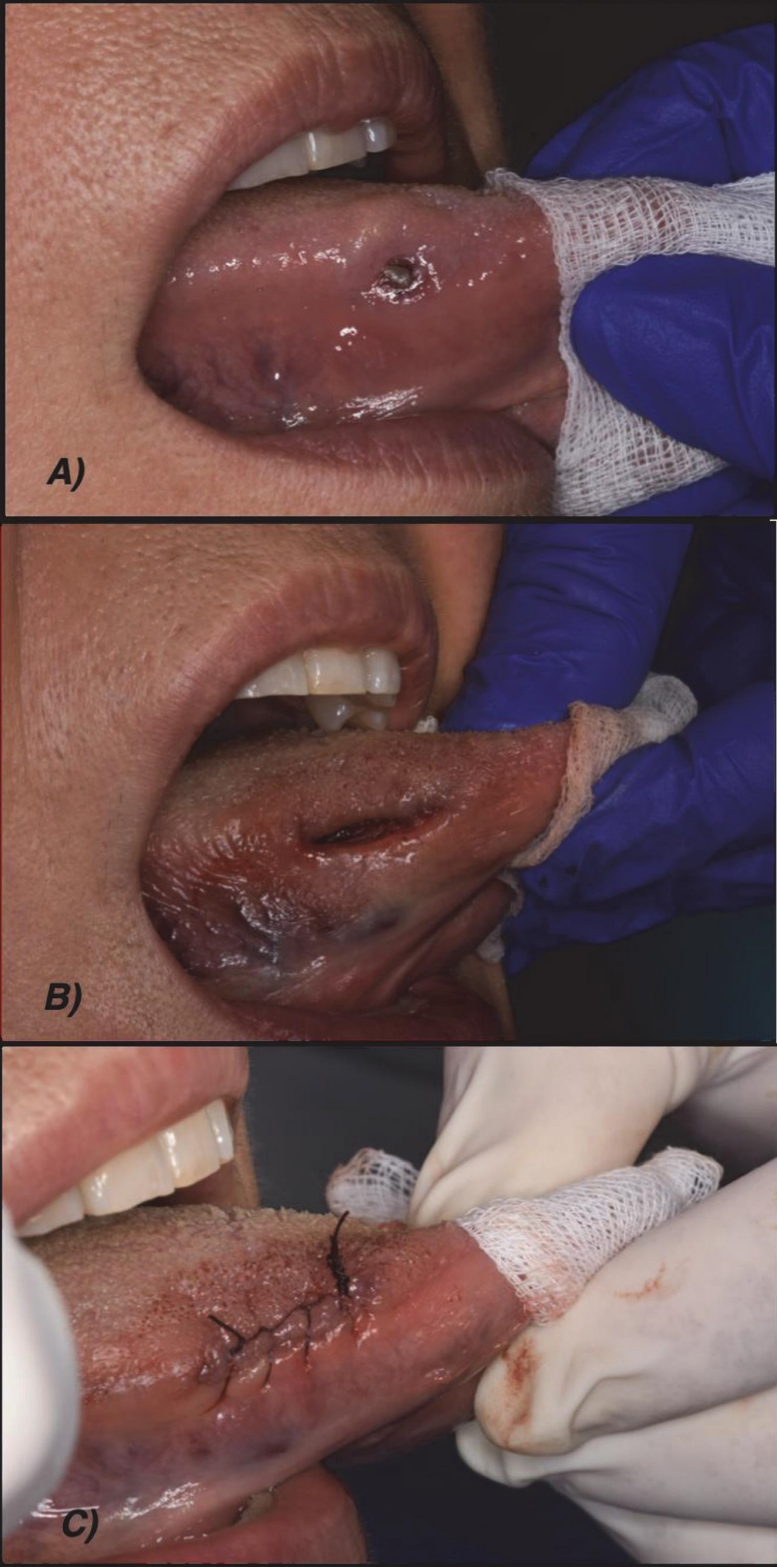
(A) Initial clinical presentation of oral EHE arising in a 58‐year‐old Italian woman; (B and C) surgical excision.

In October 2022, a 58‐year‐old woman was referred to the Department of Oral Medicine at “Policlinico Universitario Agostino Gemelli” Hospital for evaluation of a painless ulcerative lesion located on the right lateral border of the tongue, which had been progressively increasing in size. The patient’s medical history was unremarkable. Intraoral examination revealed a soft ulcerated mass with hypertrophic margins and a yellowish base measuring ~2 cm × 3 cm on the right lateral margin of the tongue (Figure 1). The patient reported that the lesion had first appeared about 1 month earlier.

**Figure 2:**
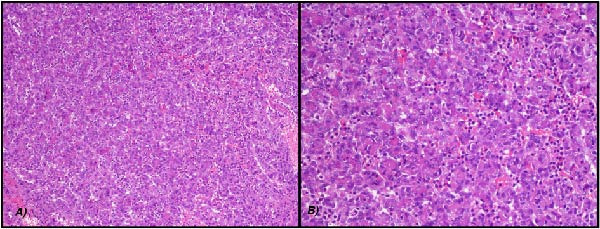
Hematoxylin and eosin staining: (A) 40x; (B) 100x.

An incisional biopsy was performed. Histopathological examination showed hyperplastic epithelium and an underlying connective tissue characterized by the proliferation of small‐caliber vascular structures composed of epithelioid endothelial cells. The stroma was infiltrated by lymphocytes, plasma cells, and eosinophils, with focal fibrinoid deposits (Figure 2). No evidence of necrosis or significant mitotic activity was observed.

Immunohistochemical analysis demonstrated positivity for ERG and negativity for S‐100, SOX‐10, HHV8, inhibin, and AE1/AE3, supporting the diagnosis of EHE.

The patient subsequently underwent surgical excision of the lesion with widening of the margins. Histological evaluation of the surgical specimen confirmed findings consistent with the initial biopsy and demonstrated clear surgical margins.

The patient was referred to the Oncology Department for systemic oncologic follow‐up, while regular intraoral clinical examinations were carried out at our department to detect possible local recurrence.

At 24 months of follow‐up after surgery, no evidence of local recurrence or distant metastasis has been observed.

### 2.2. Systematic Review

The present systematic review was conducted in accordance with the PRISMA (Preferred Reporting Items for Systematic Reviews and Meta‐Analyses) guidelines.

### 2.3. Eligibility Criteria

Studies were considered eligible if they met the following criteria: full‐text articles published in English; observational clinical studies, including case reports, case series, retrospective and prospective studies (cohort or case‐control), as well as randomized clinical trials; and studies reporting patients diagnosed with oral EHE. No specific exclusion criteria were applied.

### 2.4. Search Strategy and Study Selection

A comprehensive electronic search was performed in Medline (via PubMed), Scopus, and the Cochrane Central Register of Controlled Trials (CENTRAL) from database inception to June 2024. The last search was conducted on June 1, 2024. The search strategy combined MeSH terms and free‐text keywords related to EHE and the oral cavity, including: “epithelioid hemangioendothelioma,” “epithelioid hemangioendotheliomas,” “hemangioendotheliomas, epithelioid” in combination with “oral,” “oral cavity,” “cavity, oral,” and “cavitas oris.”

In addition, a manual search was carried out in the following journals: Oral Oncology; Clinical Oral Investigations; Journal of Oral Pathology and Medicine; Oral Surgery, Oral Medicine, Oral Pathology, and Oral Radiology; Head and Face Medicine; and Oral Diseases. The reference lists of all included articles were also screened to identify additional relevant studies.

Two reviewers (Gioele Gioco and Enrico Russo) independently screened the records using predefined data extraction forms. Titles and abstracts were first evaluated, and potentially eligible articles were subsequently assessed in full text. Only studies fulfilling the inclusion criteria were included in the review. In cases of disagreement between the two reviewers, a third reviewer (Carlo Lajolo) was consulted to reach a final decision.

### 2.5. Data Extraction and Analysis

Data regarding the main characteristics of the included studies were extracted and recorded in standardized data extraction forms. For each reported case of oral EHE, the following variables were collected: year of publication, patient age and sex, lesion size and clinical appearance, intraoral location, treatment modality, recurrence and time to recurrence, presence of metastasis, follow‐up duration, histopathological features, and immunohistochemical findings. Missing data were not considered and were therefore excluded from the analysis.

All collected data were analyzed using statistical software (SPSS Version 21.0; IBM Corp., Chicago, IL, USA).

## 3. Results

### 3.1. Results of Search and Study Selection

The initial search strategy identified a total of 82 records. After the screening and eligibility assessment process, 35 studies met the inclusion criteria and were included in the present systematic review.

**Figure 3:**
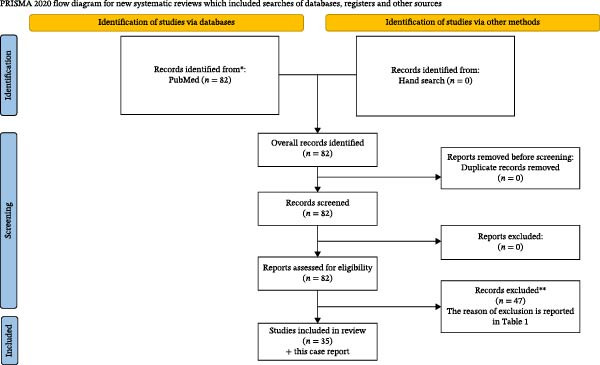
PRISMA flowchart of the systematic review.  ^∗^Consider, if feasible to do so, reporting the number of records identified from each database or register searched (rather than the total number across all databases/registers).  ^∗∗^If automation tools were used, indicate how many records were excluded by a human and how many were excluded by automation tools. *Source:* Page MJ, McKenzie JE, Bossuyt PM, Boutron I, Hoffmann TC, Mulrow CD, et al. The PRISMA 2020 statement: an updated guideline for reporting systematic reviews. BMJ 2021;372:n71. doi: 10.1136/bmj.n71. For more information, visit: http://www.prisma-statement.org/.

The selection process, reported according to the PRISMA guidelines, is illustrated in Figure 3. A summary of the full‐text articles excluded, and the reason for their exclusion is presented in Table 1.

**Table 1:** Articles excluded from systematic review and reasons for their exclusions.

Author	Year	Reason for exclusion
Liu X	2024	Nonoral EHE
Nisamudeen F	2023	Nonoral EHE
Burke AB	2024	Gorham‐Stout disease
Panda S	2023	Spindle cell hemangioma
Savithri V	2022	Spindle cell hemangioma
Liu YT	2022	Epithelioid angiosarcoma
Zhang X	2022	Nonoral EHE
Anevlavis S	2022	Nonoral EHE
Kimura H	2021	Nonoral EHE
Frezza AM	2021	Nnonoral EHE
Xia RH	2020	Primary pseudomyogenic hemangioendothelioma
Gil F	2019	Angiolymphoid hyperplasia with eosinophilia
Heng‐Maillard MA	2019	Nonoral EHE
Goss JA	2018	Review
Ennouhi MA	2018	Nonoral EHE
Hettmer S	2017	Nonoral EHE
Kang HJ	2017	Nonoral EHE
Rawal YB	2017	Pseudomyogenic hemangioendothelioma
Dutta M	2016	Papilloangioma
Sancheti S	2016	Nonoral EHE
Samuk I	2016	Nonoral EHE
Gurung S	2015	Hepatic epithelioid hemangioendothelioma
Cohen PR	2015	Review
Saada E	2014	Nonoral EHE
Li H	2014	Nonoral EHE
Cohen EE	2012	Nonoral EHE
Sangro B	2012	Nonoral EHE
Madhusudhan KS	2010	Nonoral EHE
Singhal S	2009	Nonoral EHE
Sun ZJ	2009	Epithelioid angiomatous nodule
Fleta‐Asín B	2009	Facial acneiform rash
Sandford NL	2009	Budd‐Chiari syndrome
Chatelain B	2009	Article in French
Woodall CE	2008	Hepatic malignant epithelioid hemangioendothelioma
Sheehan M	2007	Spindle cell hemangioma
Tosios KI	2008	Spindle cell hemangioma
Tucci E	2006	Bacillary angiomatosis
Mascarenhas RC	2004	Nonoral EHE
Ricalde P	2003	Vanishing bone disease
Molina Palma MI	2002	Article in Spanish
Nayler SJ	2000	Composite hemangioendothelioma
Pokharna RK	1997	Nonoral EHE
Tosios K	1995	Spindle‐cell hemangioendothelioma
Williams SB	1995	Nonoral EHE
Gelin M	1989	Nonoral EHE
Dean PJ	1985	Nonoral EHE
Ellis GL	1986	Nonoral EHE

Abbreviation: EHE, epitheliod hemangioendothelioma.

### 3.2. Study Characteristics and Summary of Results

Including the present case report, a total of 44 patients with a confirmed diagnosis of oral EHE were analyzed across the 35 selected studies.

No significant gender predominance was observed, with an equal distribution between males and females (21 men and 21 women). The mean age of the patients was 35 years (±19.17). The gingiva was the most commonly affected intraoral site, followed by the tongue (18 and 11 cases, respectively).

Clinically, the most frequent presentation was a solitary painless mass, followed by ulcerative lesions. Lesion size ranged from 0.2 to 7.0 cm, with a mean diameter of 1.7 cm. Radiographic evaluation showed that ~25% of the cases were associated with resorption or destruction of the underlying bone.

On average, the diagnosis of EHE was established ~7 months (±6.95) after the initial appearance of the lesion. Following surgical excision, recurrence occurred in 18% of cases (8 out of 44), with a mean time to recurrence of 21 months (±29.28).

Only one case of metastatic spread was reported in the literature, occurring 48 months after diagnosis and involving lymph nodes in the jugulo‐digastric and submandibular regions.

Detailed information regarding the reported cases of oral EHE, including patient age, sex, lesion location and size, presence of single or multiple lesions, possible cutaneous involvement, histopathological and immunohistochemical findings, follow‐up duration, and recurrence, are summarized in Table 2.

**Table 2:** Characteristics of patients affected by EHE.

ID case	Author	Year	Gender	Age	Clinic aspect	Clinical suspicion	Radiological features	Symptoms	Site	Size	Recurrence	Metastasis
1	Weerakoon	2021	M	23	Lump	/	—	Painless	Left border of the tongue	1 cm × 1 cm	No	No
2	Cirkin	2023	M	55	Lump	/	—	Pain, and limitation of motion	Anterior midline of the tongue	1.4 cm × 1 cm	No	No
3	Koutlas	2022	F	21	Nodule	/	—	Painless	Left mandibular vestibular mucosal	1 cm × 1 cm	No	No
4	Marrogi et al.	1991	M	45	Erythematous mass	Pyogenic granuloma	—	/	Right maxillary palatal gingiva	1.5 cm × 1 cm	Yes (3 months)	No
5	Marrogi et al.	1991	F	36	Nodule	Fibrous scar	—	Painful	Right lateral border of the tongue	0.2 cm	No	No
6	Chi et al.	2005	F	23	Located in extraction site of left mandibular third molar	/	Well defined radiolucency in the extraction site of the left mandibular third molar	Asymptomatic	Left posterior mandible	2 cm × 1.5 cm	No	No
7	Chi et al.	2005	F	28	Erythematous mass	/	Alveolar bone loss between the left maxillary canine and first premolar	Painless	Anterior maxillary gingiva	0.6 cm	No	No
8	de Araujo et al.	1987	M	4	Swelling, ulcerated lesion, tooth mobility	/	Alveolar bone resorption	/	Anterior mandibular gingiva	/	No	No
9	Flaitz	1995	F	7	Exophytic mass was described as having a bosselated, vascular appearance with focal ulceration	Pyogenic granuloma, peripheral giant cell granuloma, and peripheral ossifying fibroma with mucosal ulceration	—	Painless	Labial and lingual attached gingiva	1.5 × 0.5	No	No
10	Sun et al.	2007	M	12	Ulcerated mass, tooth mobility	/	Alveolar bone destruction	Painless	Maxillary gingiva	3 cm	No	No
11	Sun et al.	2007	M	53	Swelling, nontender mass	/	—	Painless	Buccal mucosa	1.5 cm	Yes (9 months)	No
12	Sun et al.	2007	M	17	Mild tenderness mass	/	—	/	Tongue	0.5 cm	No	No
13	Sun et al.	2007	F	52	Swelling, purple mass	/	—	/	Upper lip	2 cm	No	No
14	Sun et al.	2007	M	21	Reddish mass	/	—	/	Tongue	0.5 cm	No	No
15	Sun et al.	2007	M	34	Rubbery mass	/	—	/	Tongue	1 cm	No	No
16	Sun et al.	2007	M	11	Swelling, mass, tooth mobility	/	—	Painful	Mandibular gingiva	2 cm	No	No
17	Sun et al.	2007	M	46	Reddish firm mass	/	Alveolar bone destruction	/	Tongue	1.2 cm	Yes (4 months)	No
18	Sun et al.	2007	M	6	Reddish mass	/	—	Painful	Floor of mouth and tongue	7 cm	/	No
19	Orsini et al.	2001	F	18	Round, smooth‐surfaced, exophytic, firm, slow growing mass	/	—	Painless	Left buccal mucosa	2 cm × 1.5 cm	Yes (9 months)	No
20	Pigadas et al.	2000	F	48	Swelling, semifixed, firm, nontender mass	/	—	Intermittent sharp pain not related to eating	Right parotid salivary gland	1 cm	No	No
21	Mariatos et al.	1999	F	30	Mobile nodule	/	—	/	Vermion border of the upper lip on the right side	1 cm × 0.6 cm × 0.4 cm	/	No
22	Machalka et al.	2003	M	65	Swelling at the anterior region of jaw, gradual chin enlargement, tooth mobility	/	Poorly defined unilocular radiolucency with scattered radiopacities	Painful	Anterior mandible, recurrence extended to the right mandibular body and angle	1 cm × 1 cm	Yes (48 months)	No
23	Baehner et al.	2003	/	49	Submucosal mass	/	—	/	Lip	0.4 cm	No	No
24	Baehner et al.	2003	/	60	Mass	/	—	/	Lateral border of the tongue	1.5 cm	No	No
25	Hamakawa et al.	1999	F	76	Rubbery hard submucosal mass, mild tenderness	/	Ill‐defined radiolucency with labial cortical expansion and resorption	No spontaneous pain	Anterior mandible and anterior mandibular vestibule	4.5 cm × 4.0 cm	No	No
26	Mohtasham et al.	2008	M	9	Ulcerated reddish swelling	Pyogenic granuloma	—	Asymptomatic, painless	Maxillary gingiva	1 cm × 1 cm	Yes (12 months)	No
27	Yoruk et al.	2008	F	44	Firm mass	/	—	Painless until 6 month before. Noticed the mass ~1 year earlier	Left submandibular region (left submandibular gland)	2.0 cm × 3.0 cm	No	No
28	Cheng et al.	2007	F	13	Gengival swelling	Granulomatous inflammation	—	/	Left maxillary gingiva	/	Yes (3 months)	No
29	Manjunatha et al.	2009	M	20	Nontender with soft to firm in consistency and erythematous, pedunculated mass, tooth mobility	Peripheral giant cell granuloma	Erosion of underlying alveolar bone	Complaint of difficulty in speech	Lower lingual gingiva in the midline and floor of the mouth	3.0 cm × 4.0 cm	/	No
30	Robinson et al.	2014	F	52	An ulcerated pedunculated growth, firm, movable and crossed the vermillion border	Pyogenic granuloma or irritation fibroma, condyloma, melanoma, and squamous cell carcinoma	—	Painless	Left lower lip	2.7 cm	No	No
31	Gordón–Núñez et al.	2010	F	17	Swelling, pink, exophytic, firm, pediculated rapid growth	Pyogenic granuloma	—	Painless	Mandibular gingiva	2 cm	No	No
32	Amer et al.	2011	M	2 m	Firm mass with smooth surface at the anterior aspect of the maxilla and cheek with no displacement	/	—	/	Maxillary sinus, extending to floor of the orbit and soft tissues of the cheek	/	No	No
33	Ali et al.	2015	F	23	A small area of erythematous gingival swelling with localized bone loss around the lower anterior teeth	/	Radiographs showed horizontal and vertical bone loss around lower left incisors teeth	/	Mandibular gingiva	/	Yes (84 months)	No
34	Tong	2005	F	81	Firm submucosal mass in the right buccal region	/	—	Painless	Buccal mucosa	2 × 2	/	/
35	Bhattacharya et al.	2015	M	16	Solitary well‐defined sessile, proliferative, roughly oval growth	Pyogenic granuloma	Intraoral periapical radiograph of maxillary right first and second molars showed a homogeneous soft tissue density extending from maxillary right first molar to maxillary right second molar with no evidence of calcifications. Orthopantomograph also revealed similar findings with intact maxillary sinus floor	Discomfort with a bleeding episode from the growth	Right maxillary gingiva	3.5 cm × 3 cm	No	No
36	Sreenivasan et al.	2015	M	48	A small sessile grayish pink growth on the buccal and palatal gingiva. The swelling was firm and nontender on palpation	Pyogenic granuloma	The underlying bone showed no evidence of erosion on radiographic examination	Painless	Anterior maxillary palatal gingiva	0.7 cm × 0.7 cm	No	No
37	Salgarelli et al.	2016	M	33	Soft mucosal formation, gingival recession (Miller’s class III)	/	Mandibular radiolucency between lateral incisor and second premolar roots. Cortical bone destruction	Painless	Mandibular gingiva	1.5 cm	No	Lymphatic involvement in the left jugular‐digastric and submandibular regions after 4 years
38	Heera et al.	2017	M	46	Sessile, with an ulcerated, friable surface swelling	Pyogenic granuloma	Radiographic imaging ruled out erosion of the underlying bone	Bleeding	Posterior aspect of left side hard palate	2 cm × 2 cm × 2 cm	No	No
39	Olsen et al.	2019	F	73	Ulcerated gingival lesion (associated with tooth 4.4 with adjacent horizontal loss of bone)	/	Horizontal alveolar bone loss	Asymptomatic	Mandibular gingiva	/	No	No
40	Yang et al.	2019	M	44	Numbness of the left lower lip and painful swell of the left mandible–slight lingual expansion of the left ascending ramus of the mandible	/	Osteolytic lesion in the angle and ramus of the mandible, with lingual cortical destruction but no periosteal reaction	Painful	Lower lip and left mandible angle and ramus of the mandible	4.5 cm × 3.5 cm × 1 cm	No	No
41	Rajendrakumar et al.	2019	F	41	Red, ulcerated, friable, and progressively enlarging mass	Pyogenic granuloma	Showed bone resorption in the upper and lower anterior region	Painless and on palpation there was associated bleeding	Labial gingiva of maxillary arch from right second premolar to left lateral incisor extending over the hard palate	3 cm × 4 cm	No	No
42	Januzis et al.	2019	F	18	Palatal gingiva were recessed leaving exposed the fist premolar and canine. Canine and both premolars had second‐grade mobility	Marginal periodontitis	Bone destruction in the defect area reaching the maxillary sinus, whose mucosa was locally thickened	/	Right maxillary palatal gingiva	/	No	No
43	Yendluri et al.	2023	F	6	Solitary growth, sessile with a yellowish with surface, ulcerated borders	Fibroma	—	/	Ventrolateral surface of tongue	1.5 × 0.7	No	No
44	Gioco et al.	2024	F	58	Single, round, well‐demarcated ulcer with hypertrophic elevated margins and yellowish base	/	—	Painless	Right lateral border of the tongue	0.7 cm × 0.5 cm	No	No

## 4. Discussion

Vascular lesions encompass entities with a proliferative ability ranging from local growth, disfigurement, and local recurrence to distant metastases. The International Society for the Study of Vascular Anomalies (ISSVA) has developed guidelines to further characterize vascular tumors based on their malignant potential. In 2018, the vascular tumors class was expanded, and many new rare entities were added. Three macro‐classes were defined: benign, locally aggressive or borderline, and malignant [10].

The term hemangioendothelioma was introduced by Borrmann, who first proposed the concept of vascular neoplasms with intermediate or low malignant potential [11].

The hemangioendotheliomas tumor is characterized by endothelial cell proliferation around a vascular lumen. Moreover, hemangioendotheliomas are further classified into three main histopathologic types: kaposiform, hobnail (or Dabska‐retiform), and epithelioid. The kaposiform type are characterized by infiltrating, rambling nodules of small, round capillary hemangioma‐like vessels intermixed with slit‐like Kaposi sarcoma‐like vessels. The hobnail hemangioendotheliomas exhibit hobnail or cuboidal, apically ‘‘bulging” endothelial cells [12].

The epithelioid type was first described by Weiss and Enzinger [3] in 1982 as a vascular neoplasm of borderline malignant potential. Histologically, it is characterized by a proliferation of rounded, eosinophilic endothelial cells with frequent cytoplasmic vacuolization with a distinctive epithelioid appearance and a tendency of angiocentricity. The growth pattern into a myxohyaline stroma potentially leading to a mistaken diagnosis of carcinoma [13]. It can occur in the superficial or deep soft tissue of extremities, liver, and bone. The clinical presentation of EHE is heterogeneous: 30% of cases presenting as pulmonary EHE. Other reported sites include liver (21%), liver plus lung (18%), lung alone (12%), and bone alone (14%), and few oral involvements are reported [14].

This systematic review retrieved a total of 44 cases of oral EHE. Even if few cases are reported in literature, some consideration should be performed regarding the main differences between oral and extra‐oral EHE.

Clinically, oral EHE can have a wide range of presentation and it could affect the entire oral mucosa. The gingiva is the most common site, followed by the tongue, maxillary and buccal mucosa, and palate and lip, respectively [15]. The most common presentation of EHE is a painless solitary mass appearing erythematous, purple‐pink to yellowish in color. Localized swellings with or without ulceration, pedunculated or sessile, or growth may be the primary manifestation of the lesion [16]. A good number of cases presenting with a bleeding friable soft tissue mass. The size of lesions ranged from 0.2 to 7.0 cm, with a mean of 1.7 cm. The analysis of radiographic features showed that 25% of the lesions were associated with resorption or destruction of underlying bone, highlighting the need for careful radiographic examination of the lesions to investigate possible alterations of the bone.

Radiological assessment may represent a useful adjunct in the evaluation of EHE, particularly to assess the local extension of the lesion and to exclude bone involvement or distant disease. However, due to the rarity of this tumor and the limited number of reported oral cases, no standardized imaging protocols or evidence‐based recommendations regarding the most appropriate radiological modality currently exist. When deeper soft tissue extension is suspected, magnetic resonance imaging (MRI) may be helpful to better delineate lesion margins and its relationship with adjacent structures, whereas computed tomography (CT) may be useful in cases with suspected bone involvement. Only a limited number of studies included in this review reported radiological assessment (Table 2).

Histologically, EHE is characterized by nests and cords of endothelial cells with epithelioid morphology embedded within a fibrotic or hyalinized stroma, often showing cytoplasmic vacuolization. In some cases, areas with spindle‐shaped cells and small capillary‐like vascular channels may also be observed [17]. Features such as cytological atypia, cellular pleomorphism, high mitotic activity, a higher proportion of spindling cells than epithelioid cells, focal necrosis, metaplastic bone formation within the tumor, as well as numerous osteoclast‐like giant cells, indicate potential and increased malignant and aggressive behavior [18].

Immunohistochemistry plays a key role in confirming the diagnosis, as tumor cells typically express endothelial markers such as CD31, ERG, CD34, and factor VIII‐related antigen, helping to differentiate EHE from other vascular or soft tissue lesions [19–24].

Considering the differential diagnosis, it can clinically mimic, depending on the site, periodontitis [25], cystic lesions (epidermoid/dermoid, deep‐seated mucoceles, and foregut cysts), histiocytosis (histiocytosis X and oral juvenile xanthogranuloma) [26], and benign or vascular tumors (fibromas, peripheral ossifying fibroma, leiomyoma, rhabdomyoma, neurofibroma, amelanotic melanoma, pyogenic granuloma, peripheral giant cell granuloma, inflammatory fibrous hyperplasia, and necrotizing ulcerative gingivitis) [27–34].

The histological differential diagnosis includes both benign and malignant entities: squamous cell carcinoma, epithelioid angiosarcoma, epithelioid sarcoma, hemangiopericytoma, hemangioma, Kaposi sarcoma, metastatic carcinoma, and adenocarcinoma [35, 36].

The main differential diagnosis should be conducted with epithelioid angiosarcoma; thus, it represents the malignant counterpart of EHE. The presence of less pleomorphism, less mitotic activity, and less cytological atypia in EHE aids differentiation from epitheliod angiosarcoma. Also, metastatic carcinoma and squamous cell carcinoma are characterized by higher mitotic activity, with a plethora of cytological atypia and pleomorphism. Other entities should be considered in the differential diagnosis. Kaposi sarcoma can be excluded due to the absence of epithelioid cells with intracytoplasmic vacuoles and the presence of fibrosarcoma‐like spindle cells in the EHE lesions. In hemangiopericytoma, the tumor cells are positive for SMA and exclude the diagnosis of EHE.

Due to the morphological appearance of epithelioid tumor cells of EH and the fact that many lesions are reactive for cytokeratin (AE‐1/AE‐3), it requires great care on the part of the pathologist to avoid the risk of misdiagnosis of squamous cell carcinoma. Variable immunohistochemical expression of SMA has previously been observed for intraoral EHEs. The nuclear transcription factor Fli‐1 (a specific marker for Ewing’s sarcoma and primary neuroectodermal tumor) was found to be sensitive to vascular lesions, including EHE.

Surgical excision with adequate margins remains the primary treatment for EHE; however, due to the rarity and variable clinical course of this tumor, no standardized management guidelines have been established. In literature, the recurrence rate is about 20% [37]. These findings suggest that intraoral EHEs are less aggressive in nature. Given the possibility of recurrence and metastasis several years after clean and safe excision, clinicians should apply an adequate follow‐up period for this malignancy (at least 5 years).

Systemic metastases have been described in the literature at a rate of 21%, while a mortality rate of 17% has been described for cutaneous EHE, in agreement with the histological aspects of the neoplasm. Regarding the oral cavity, only one case of metastasis from gingival EHE with lymphatic involvement in the jugulo‐digastric and submandibular regions has been reported in the literature [38]. The metastasis occurred 48 months after diagnosis. It is therefore important that the follow‐up clinical examination includes the evaluation of regional lymph nodes. Moreover, distinguishing between multicentric and metastatic disease represents a critical aspect in the evaluation of EHE. This distinction generally relies on a comprehensive clinical assessment combined with radiological imaging aimed at excluding distant disease. PET‐CT should be incorporated into the diagnostic workup when multicentric disease is suspected and during follow‐up. The development of new lesions should be regarded as indicative of metastatic disease. In the present case, the patient underwent local clinical follow‐up for the detection of possible local recurrence, while systemic oncologic evaluation and surveillance were performed by the Oncology Department.

Nevertheless, follow‐up strategies for EHE remain poorly defined due to the rarity of the disease and the limited number of reported cases. The available evidence largely consists of isolated case reports and small case series; consequently, robust evidence‐based recommendations and standardized management guidelines are currently lacking.

## 5. Conclusion

The differential diagnosis of oral soft tissue swellings should also consider vascular tumors, which may pose a diagnostic challenge due to their clinical resemblance to several other lesions, including cysts, reactive lesions, and benign neoplasms. Although this condition is rare, the diagnostic process should begin with a careful evaluation of the patient’s medical history and a thorough clinical examination. However, definitive diagnosis requires histopathological evaluation supported by immunohistochemical analysis. The recommended management generally consists of wide local surgical excision with adequate safety margins. Furthermore, patients diagnosed with EHE should undergo long‐term follow‐up, given the risk of local recurrence and the tumor’s uncertain malignant potential.

## Author Contributions

Conceptualization: Carlo Lajolo and Gioele Gioco. Methodology: Romeo Patini. Validation: Carlo Lajolo, Romeo Patini, Gioele Gioco, and Cosimo Rupe. Formal analysis: Gioele Gioco and Enrico Russo. Data curation: Cosimo Rupe. Writing – original draft preparation: Gioele Gioco, Enrico Russo, and Carlo Lajolo. Writing – review and editing: Guido Rindi and Carlo Lajolo. Visualization: Guido Rindi and Carlo Lajolo. Supervision: Gioele Gioco.

## Funding

Open access publishing facilitated by Universita Cattolica del Sacro Cuore, as part of the Wiley ‐ CRUI‐CARE agreement.

## Disclosure

All authors have read and agreed to the published version of the manuscript.

## Ethics Statement

The authors have nothing to report.

## Consent

All the patients allowed personal data processing, and informed consent was obtained from all individual participants included in the study.

## Conflicts of Interest

The authors declare no conflicts of interest.

## Supporting Information

Additional supporting information can be found online in the Supporting Information section.

## Supporting information


**Supporting Information** File S1: CARE checklist.

## Data Availability

The data that support the findings of this study are available from the corresponding author upon reasonable request.
