# Supplementary material for: A Rare Epithelioid Hemangioendothelioma of the Tongue: A Case Report and Review of Published Cases
Source: Case Rep Dent. 2026 May 22;2026:4933968. doi: 10.1155/crid/4933968 (PMC13195496; doi:10.1155/crid/4933968)
Supplement: Supplementary file 1 — Supporting Information File S1: CARE checklist. [file CRID-2026-4933968-s001.docx]

**Supplementary File 1**. CARE Checklist
